# Comparison of the early clinical efficacy of the SuperPath approach versus the modified Hardinge approach in total hip arthroplasty for femoral neck fractures in elderly patients: a randomized controlled trial

**DOI:** 10.1186/s13018-023-03713-9

**Published:** 2023-03-19

**Authors:** Jiquan Shen, Weiping Ji, Yonghui Shen, Shijie He, Youbin Lin, Zhijun Ye, Bo Wang

**Affiliations:** 1 Department of Orthopaedics, The Sixth Affiliated Hospital of Wenzhou Medical University, The People’s Hospital of Lishui, Lishui, 323000 Zhejiang China; 2 Department of Orthopaedics, The People’s Hospital of Yunhe, Lishui, 323000 Zhejiang China

**Keywords:** Total hip arthroplasty, SuperPath approach, Modified Hardinge approach, Early clinical efficacy, Learning curve

## Abstract

**Purpose:**

To investigate the clinical efficacy and advantages of the SuperPath approach for total hip arthroplasty in the treatment of femoral neck fractures in the elderly population.

**Methods:**

From February 2018 to March 2019, 120 patients were randomly divided into two groups with 60 patients each: the SuperPath group and the conventional group. The results evaluated included the general operation situation, serum markers, blood loss, pain score, hip function and prosthesis location analysis.

**Results:**

There was no demographic difference between the two groups. Compared with the conventional group, the SuperPath group had a shorter operation time (78.4 vs. 93.0 min, *p* = 0.000), a smaller incision length (5.8 vs. 12.5 cm, *p* = 0.000), less intraoperative blood loss (121.5 vs. 178.8 ml, *p* = 0.000), a shorter hospitalization time (8.0 vs. 10.8 days, *p* = 0.000) and less drainage volume (77.8 vs. 141.2 ml, *p* = 0.000). The creatine kinase level in the SuperPath group was significantly lower than that in the conventional group, while there was no difference in the C-reactive protein level and erythrocyte sedimentation rate level. The visual analog scale score was lower one month postoperatively, and the Harris hip score was higher three months postoperatively in the SuperPath group (*p* < 0.05). There was no difference in the cup abduction angle or anteversion angle of the two groups.

**Conclusion:**

We found better clinical efficacy after using the SuperPath approach with less muscle damage, less postoperative pain and better postoperative function than after using the modified Hardinge approach.

*Trial registration* The randomized clinical trial was retrospectively registered at the Chinese Clinical Trial Registry on 31/12/2020 (ChiCTR-2000041583, http://www.chictr.org.cn/showproj.aspx?proj=57008).

## Introduction

A femoral neck fracture is a common form of hip fracture in the elderly population, and the mortality rate is as high as 25% in the first year after the fracture [1–3]. Total hip arthroplasty (THA), one of the main treatments for femoral neck fractures, can significantly improve the quality of life of patients with end-stage hip joint disease, increase function, restore the range of joint movement, and relieve pain [4–6]. There are multiple surgical approaches for THA, including the traditional posterior approach (PA), anterior approach (AA), lateral approach (LA), anterolateral approach, posterolateral approach, and modified Hardinge approach [7–9]. These traditional surgical approaches have some common shortcomings, including external trochanter muscle injury, postoperative dislocation and a long recovery time [10–12]. The ideal approach for THA should reach the surgical site completely through the muscles, blood vessels, and nerve spaces and it has the advantages of less damage, less bleeding, fewer postoperative pain symptoms, and faster recovery, but there is currently no single approach to achieve this ideal state [13–15]. In 2011, Dr. James Chow first reported the supercapsular percutaneously assisted total hip (SuperPath) approach, which combines the advantages of the supercapsular (SuperCap) approach and the percutaneously assisted total hip (PATH) approach [12]. The SuperPath technique reaches the hip joint capsule through the tissue gap between the gluteus medius and the piriformis, so this technique no cutting any muscles and tendons [16, 17]. Previously, SuperPath technology has shown that the time to go to the ground is shortened, the hospital stay is short, the incidence of complications is low, and the in-hospital cost is low [18, 19].

Since SuperPath approach introduction, several studies were conducted to reveal differences in outcomes of SuperPath approach in comparison to conventional approaches in THA [20–29]. The conclusions of these studies are different. The 2020 meta-analysis by Ramadanov et al. [30] showed that comparing with conventional approach, SuperPath approach showed better results only in terms of decreasing incision length and early pain intensity, and there was no difference in terms of postoperative Harris hip score (HHS), acetabular cup positioning, intraoperative blood loss, hospital stay and postoperative complications, and the operation time of SuperPath approach was longer. Significantly, in 2022 meta-analysis [31], the author corrected the misinterpretation in his first THA meta-analysis by further incorporating randomized controlled trials (RCTs), increasing the overall sample size and using high-quality statistical methods. New research showed that SuperPath approach were superior conventional approach in all measured surgical and functional outcomes besides operation time. Although the SuperPath approach is receiving increased attention and application in the clinical setting, clinical research on the SuperPath approach is still insufficient, so we compared the results of the SuperPath approach with modified Hardinge approach in a randomized controlled trial. The purpose of this study was to compare the clinical efficacy of the SuperPath approach with the modified Hardinge approach in the treatment of femoral neck fractures and share some skills in SuperPath approach surgery.

## Materials and methods

### Study design

This was a prospective randomized controlled trial of patients with femoral neck fracture. The study was conducted according to the “CONSORT statement” guidelines for randomized control trials. This research was approved by the Medical Ethics Committee of the Lishui People’s Hospital, The Sixth Affiliated Hospital of Wenzhou Medical University, Lishui, China. The inclusion criteria were as follows: (1) patients with fresh femoral neck fractures (Garden tapes III and IV); (2) patients age ≥ 65 years; (3) patients with a body mass index (BMI) < 30 kg/m^2^; and (4) patients who signed informed consent forms for clinical research. The exclusion criteria were as follows: (1) patients with pathological fractures, femoral head necrosis, and arthritis of the affected hip joint; (2) patients with hip joint disease, severe hip joint anatomical deformities, and hip joint dysfunction before the fracture; (3) patients with a BMI > 30 kg/m^2^; (4) patients who were unwilling to participate in the trial; (5) patients who were treated with hemiarthroplasty and (6) cognitive impairment and dementia.

### Sample size and power considerations

The sample size for this trial is based on an expected mean difference between groups of HHS. We used the HHS 3-month after THA increased ≥ 2 as effective, with a standard deviation (SD) of 3 according to our clinical experience. The error was set at 0.05 and the power level at 90%, a minimum of 49 patients per group was needed. This minimal sample size estimate has been increased by 20% after considering the potential dropouts, finally including 59 patients for each group.

### Patients

**Fig. 1:**
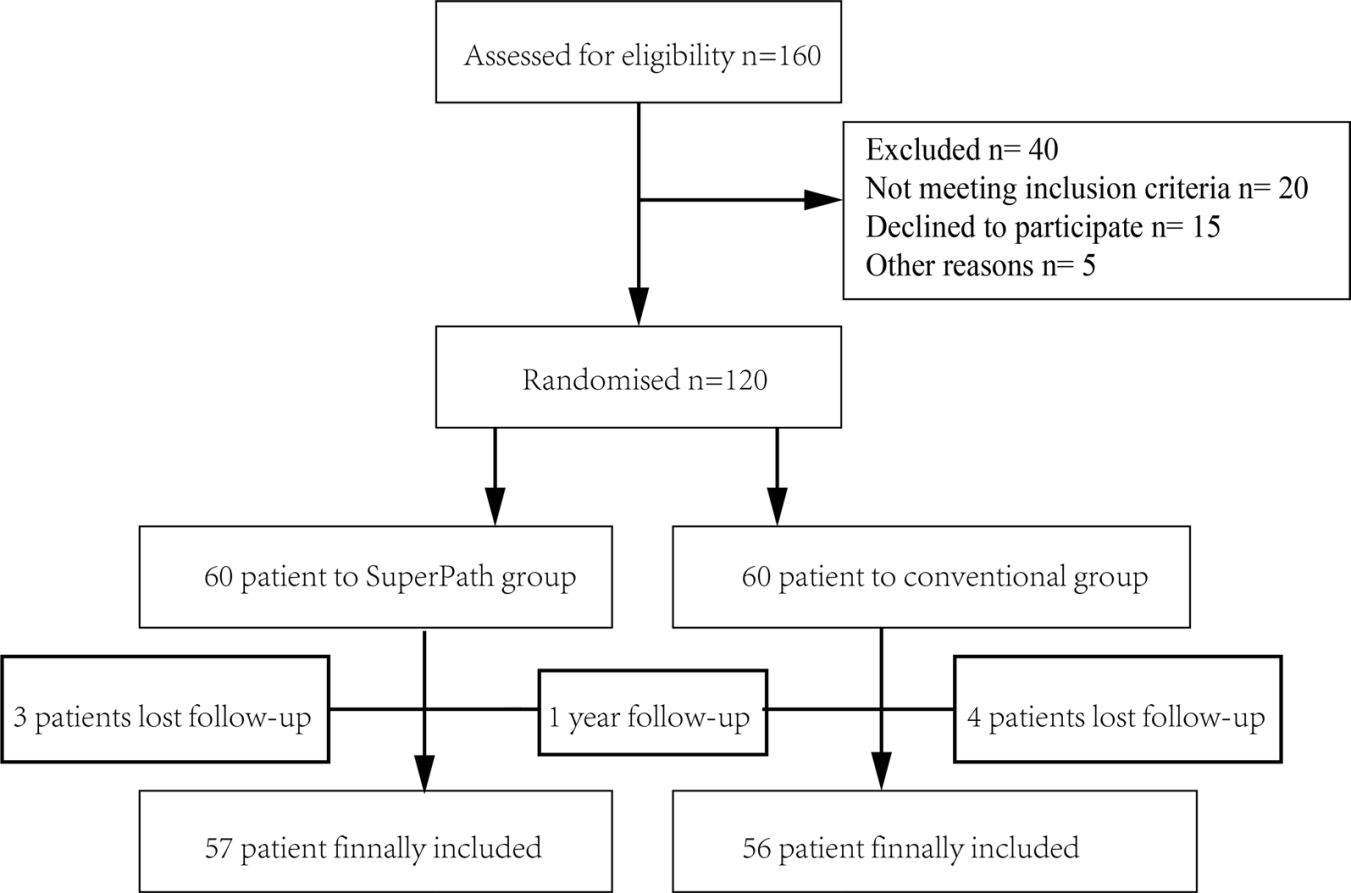
Flow diagram of the progress through the phases of the randomized trial

From January 2018 to April 2019, a total of 160 consecutive THA patients with femoral neck fractures were eligible in our hospital, of which 120 patients were enrolled and randomly divided into two groups in this study (Fig. 1). After confirmation of eligibility, patients were randomized (1:1) to receive the SuperPath or modified Hardinge approach for THA using the simple randomization method. The allocation sequence was computer-generated (http://tools.medsci.cn/rand). The study population consisted of 60 patients undergoing THA with the SuperPath approach (SuperPath group) and 60 patients undergoing THA with the modified Hardinge approach (conventional group). All operations were performed by a senior surgeon at our hospital (Complete at least 50 modified hardinge and superpath approach independently). The surgical endoprothesis were as follows: the SuperPath total hip prosthesis system was provided by MicroPort Orthopedics, Incorporated (Shanghai). The modified Hardinge total hip prosthesis system was provided by Biomet Orthopedics (USA). All implants were biological materials. This study is non-blind to researchers and patients.

### Efficacy evaluation index

All patients were followed up in the orthopedics unit of the Lishui People’s Hospital, Lishui, China. The general operation situation, serum markers, blood loss, pain score, hip function and prosthesis location analysis were evaluated between the two groups. The postoperative outcomes were assessed at 1-week, 1-month, 3-month, 6-month and 12-month follow-up intervals after the operation. All data were collected by a research fellow and a postgraduate student, not by the operating surgeon. One-year follow-up was the primary endpoint. Patient demographic characteristics were collected, including age, sex, side, body mass index, and American Society of Anesthesiologists (ASA) class. The following indicators were used to assess the functional outcome of the two groups. (1) General operation situation: operation time, intraoperative blood loss, incision length, hospitalization time and the occurrence of surgery-related complications; (2) serum markers: inflammatory response indicators [C-reactive protein (CRP) level, erythrocyte sedimentation rate (ESR) level] and muscle injury indicators [creatine kinase (CK) level]. These serum markers were collected for each patient on the day of hospital admission, and postoperative Days 1, 3, and 7. (3) Blood loss: Hemoglobin (Hb) and hematocrit (HCT) level were collected for each patient on the day of hospital admission, and postoperative days 1, and 3. Drainage tubes were routinely placed in each patient, the drainage tube was removed and the drainage volume was recorded on the first postoperative day. (4) Pain and hip function: visual analog scale (VAS) scores were collected on the day of hospital admission, postoperative days 1, 3, and 7, and 1 month, 3 months, 6 months, and 12 months after surgery. The HHS was evaluated one week, 1 month, 3 months, 6 months, and 12 months postoperatively. (5) Component placement analysis: All postoperative anteroposterior pelvic radiographs were standardized with the patient in the supine position and the radiation beam was focused on the pubic symphysis with a focal length of 100 cm. The abduction angle and anteversion angle were measured on anteroposterior pelvic radiographs according to the method described by Bachhal et al. [32].

### SuperPath approach surgical technique

**Fig. 2:**
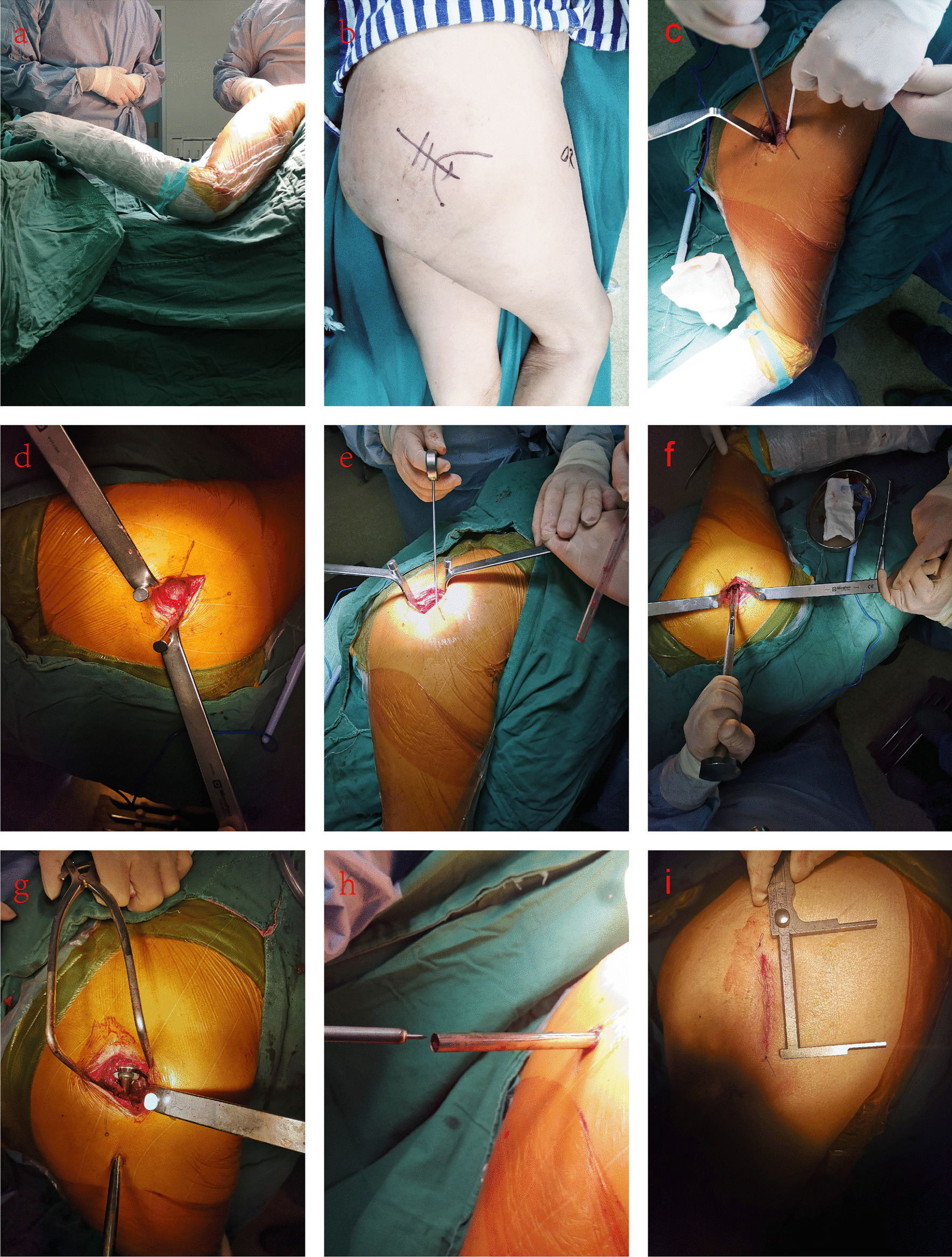
The SuperPath approach for total hip arthroplasty (THA) for femoral neck fractures: **a** Patient positioning; **b** Skin incision; **c** Split the gluteus maximus muscle and expose the posterior edge of the gluteus medius muscle; **d**Two blunt Hohmann retractors are inserted before and after the joint capsule; **e** Determine the direction of the medullary cavity; **f** Create the initial canal and ensure the anteversion angle of the femoral component; **g** Place the acetabular implant and determine the position; **h** Insert the screw; **I** Close the incision

The patient is placed in the standard lateral decubitus position with the operative hip in a slightly adducted position, with 45°–60° flexion, and 20°–30° internal rotation (Fig. 2a). A skin incision is made from the tip of the trochanter to the trochanter at 5–7 cm proximally in line with the femur (Fig. 2b). The gluteus maximus muscle is split by two wing-tipped elevator dissections in line with the fibers (or by using the index finger to separate the gluteus maximus and touching the posterior edge of the gluteus medius and the space between the piriformis and gluteus minimus); then, the posterior edge of the gluteus medius muscle is exposed with a Cobb elevator (Fig. 2c). Two blunt Hohmann retractors are inserted before and after the joint capsule (Fig. 2d). The hip capsule is then incised from the saddle of the femoral neck to 1 cm proximal to the acetabulum rim. The zenith of the femoral canal is made with a sharp starter reamer medial to the femoral trochanter apex. A femoral orientation is inserted from the zenith to the femoral condyle to determine the direction of the medullary cavity (Fig. 2e). A round calcar punch is used to remove a small portion of the bone from the outside of the femoral head for slotting to ensure the anteversion angle of the femoral component (Fig. 2f). Using the ream and broach system, the femoral metaphysis is reamed in order from small to large until resistance meets the isthmus of the femoral canal. The broach handle is then removed at the final size, leaving the broach in place to act as a femoral component trial. After raising the leg to the abduction position, the femoral neck osteotomy is performed using an oscillating saw at the top of the medullary cavity file. According to the alignment handle and guide, a 1 cm auxiliary incision is made located at the posterior edge of the femur. Using the acetabular file holder, the acetabular file is hanged into the acetabulum through the main incision and the acetabulum is filed in order of size. Acetabular basket reamers are used to ream through the main incision until an appropriately sized acetabular implant is chosen. The chosen acetabular implant is placed and the position is determined by the three screw hole indicator lines on the mortar cup aligned with the mortar crest from 11 to 1 o’clock (Fig. 2g). Screw drilling with a sleeve inside the cannula and insertion with a straight ratchet screwdriver can be achieved through the cannula when necessary (it is more difficult for a beginner to place screws. At this time, we recommend that the original MicroPort drill be replaced with a 2.0 Kirschner wire for drilling, which will make the drilling easier. Before preparing to insert the screw, you can touch the screw hole distribution position with your finger) (Fig. 2h). Then, the lining is implanted. After trial reduction, flexion (100°–120°), extension (5°–10°), internal rotation (30°–40°) and external rotation (30°–40°) of the patient’s limb is performed and C-arm fluoroscopy is used to test the stability of the trial implants. The trial implants are replaced with definitive prostheses and then stability is checked again. Finally, routine suturing of the capsule, the gluteus maximus fascia, subcutaneous tissue and skin are performed successively (Fig. 2i).

### Statistical analysis

Values are expressed as the means ± standard deviations (SDs). Differences between the two groups were performed using the independent sample *t* test, Fisher’s exact test and the *χ*^2^ test using SPSS (version 6.0; SPSS Inc., Chicago, IL, USA). Statistical analysis was performed using GraphPad Prism. Values of *p* < 0.05 were considered to be statistically significant.

## Results

All 120 patients successfully underwent the operation, and 3 patients were lost to follow-up in the SuperPath group, while 4 patients were lost follow-up in the conventional group. The patients’ demographic information is shown in Table 1. The SuperPath group comprised the following: 29 males and 28 females, aged 66 to 93 years, with an average age of 75 years; 32 cases of left hip fractures and 25 cases of right hip fractures; BMIs from 17.3 to 27.4 kg/m^2^, with an average BMI of 21.5 kg/m^2^; and ASA values of 2.07 ± 0.42. The conventional group comprised the following: 26 males and 30 females, aged 65–90 years, with an average of 74 years; 29 cases of left hip fractures and 27 cases of right hip fractures; BMIs from 17.3 to 25.9 kg/m^2^, with an average BMI of 21.7 kg/m^2^; and ASA values of 2.05 ± 0.35. There were no statistically significant differences in sex, age, fracture side, BMI, or ASA value between the two groups (*p* > 0.05).

**Table 1:** Preoperative characteristics of patients in two groups (Mean ± SD)

Parameters	SuperPath group (n = 57)	Conventional group (n = 56)	*p* Value
No. of patients	57	56	–
Age (years)	75.2 ± 6.4	74.4 ± 6.1	0.505
Gender(M/F)	29/28	26/30	0.640
Side (L/R)	32/25	29/27	0.518
BMI (kg/m^2^)	21.5 ± 2.2	21.7 ± 2.0	0.529
ASA (grade)	2.07 ± 0.42	2.05 ± 0.35	0.820

*BMI* body mass index, *ASA* American Society of Anesthesiologists

**Fig. 3:**
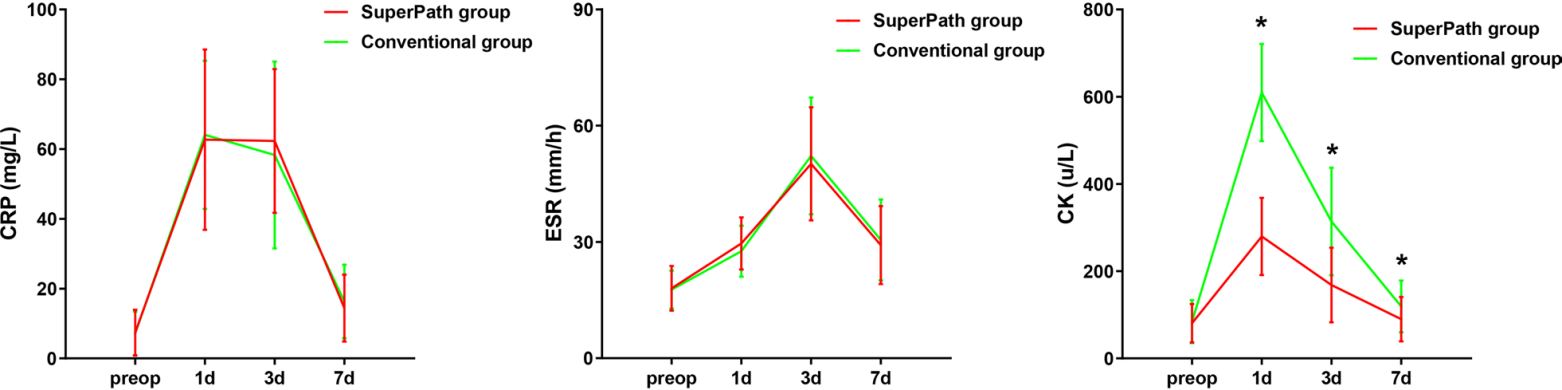
Pre- and postoperative levels of serum markers (CRP, ESR and CK) in the SuperPath and conventional groups. *Y*-axis represents levels of serum markers and *x*-axis represents denotes the time points. *A *p* < 0.05 indicates significant differences from the conventional group

**Fig. 4:**
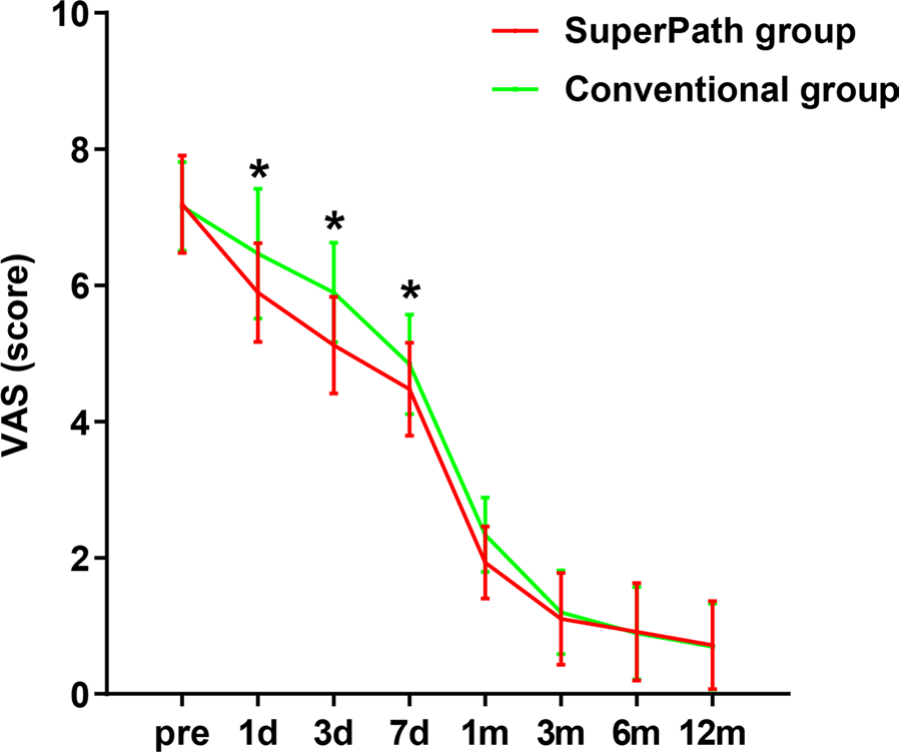
Pre- and postoperative visual analog scale (VAS) in the SuperPath and conventional groups. *Y*-axis represents VAS score and *x*-axis represents denotes the time points. *A *p* < 0.05 indicates significant differences from the conventional group

**Fig. 5:**
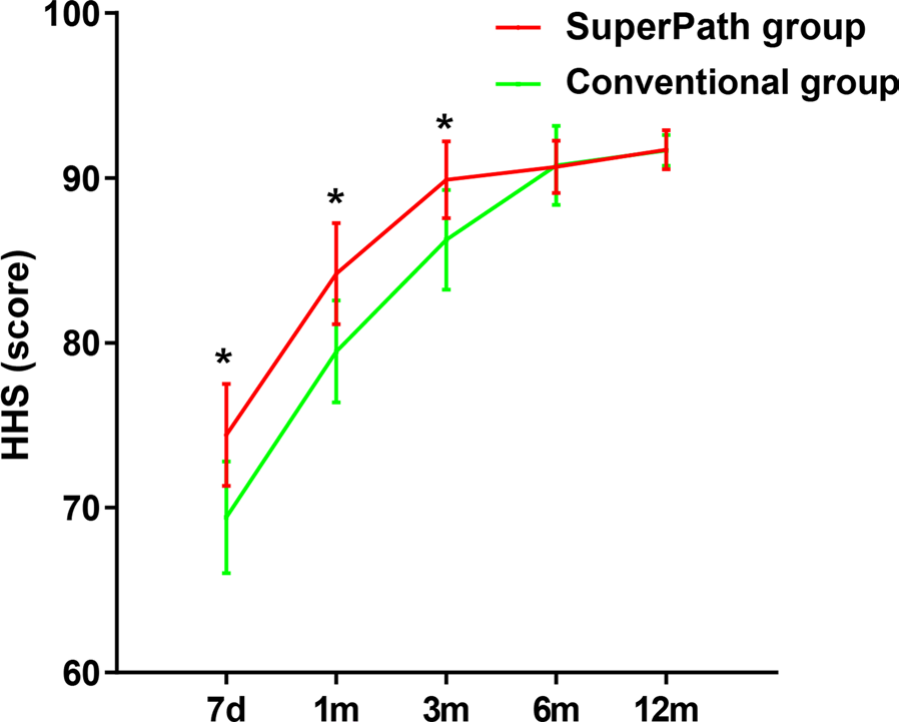
Pre- and postoperative Harris hip scores (HHSs) in the SuperPath and conventional groups. *Y*-axis represents HHSs and *x*-axis represents denotes the time points. *A *p* < 0.05 indicates significant differences from the conventional group

Compared with the conventional group, the SuperPath group had a shorter operation time, smaller incision length, less intraoperative blood loss, shorter hospitalization time and less drainage volume (*p* < 0.05). There was no significant difference in the postoperative blood transfusion rate between the two groups (Table 2). There was no significant difference in CRP and ESR levels between the two groups on the day of hospital admission, or postoperative days 1, 3, or 7 (*p* > 0.05). The CK level in the SuperPath group was significantly lower than the CK level in the conventional group at postoperative Day 1, Day 3, and Day 7 (Table 3, Fig. 3). There was no significant difference in Hb and HCT levels between the two groups on the day of hospital admission, or on postoperative Days 1, 3, and 7 (*p* > 0.05) (Table 4, Fig. 4). The trends of the HHS and VAS score in the two groups are shown in Fig. 5. There was no statistically significant difference in the VAS scores of the two groups on the day of hospital admission (*p* > 0.05). The VAS scores in the SuperPath group at the 1-, 3-, 7-day and 1-month follow-up intervals were significantly lower than the VAS scores in the conventional group, but were not significantly different at the 3-, 6- and 12-month follow-up intervals (*p* > 0.05) (Table 5). The HHS values in the SuperPath group were significantly higher than those in the conventional group at the 7-day, 1- and 3-month follow-up intervals, but were not significantly different at the 6- and 12-month follow-up intervals (*p* > 0.05) (Table 5). There was no significant difference between the cup abduction angles and anteversion angles of the two groups, and they were all within the Lewinneks’ safe zone [33] (Table 6). None of the patients had prosthesis dislocation, proximal femoral fractures, postoperative infections, nerve damage, or heterotopic ossification in the two groups.

**Table 2:** Perioperative general operation situation in two groups (Mean ± SD)

Parameters	SuperPath group (n = 57)	Conventional group (n = 56)	*p* Value
Operation time (min)	78.3 ± 8.0	93.6 ± 10.9	0.000*
intraoperative blood loss (ml)	122.3 ± 30.1	178.2 ± 31.6	0.000*
Incision length (cm)	5.8 ± 0.4	12.5 ± 0.8	0.000*
hospitalization time (days)	8.1 ± 1.3	10.8 ± 1.4	0.000*
drainage volume (ml)	77.8 ± 48.1	137.2 ± 57.0	0.000*
Transfusion rate	2/57	3/56	0.636

*p < 0.05 indicate significant differences from the conventional

**Table 3:** Perioperative serum markers change in two groups (Mean ± SD)

Parameters	Timings	SuperPath group (n = 57)	Conventional group (n = 56)	*p* Value
CRP (mg/l)	Pre	7.39 ± 6.55	7.16 ± 6.27	0.848
	1 d	62.72 ± 25.83	64.10 ± 21.27	0.757
	3 d	62.34 ± 20.58	58.31 ± 26.81	0.371
	7 d	14.45 ± 9.63	16.33 ± 10.51	0.323
ESR (mm/h)	Pre	18.04 ± 5.75	17.68 ± 4.94	0.725
	1 d	29.60 ± 6.72	27.63 ± 6.61	0.119
	3 d	50.16 ± 14.61	52.18 ± 15.07	0.471
	7 d	29.19 ± 10.06	30.52 ± 10.45	0.494
CK (u/l)	Pre	80.70 ± 43.90	84.70 ± 49.30	0.650
	1 d	279.58 ± 88.67	609.71 ± 111.31	0.000*
	3 d	168.54 ± 85.61	314.02 ± 123.19	0.000*
	7 d	89.95 ± 50.90	119.14 ± 59.27	0.006*

*p < 0.05 indicate significant differences from the conventional. *CRP* C-reactive protein, *ESR* erythrocyte sedimentation rate, *CK* creatine kinase

**Table 4:** Perioperative hemoglobin and hematocrit change in two groups (Mean ± SD)

Parameters	Timings	SuperPath group (n = 57)	Conventional group (n = 56)	*p* Value
Hb (g/L)	Pre	122.2 ± 17.4	124.7 ± 14.8	0.415
	1 d	98.5 ± 12.0	97.3 ± 12.7	0.615
	3 d	96.4 ± 10.9	94.6 ± 12.3	0.423
HCT (%)	Pre	36.4 ± 5.3	37.2 ± 4.2	0.369
	1 d	29.4 ± 3.8	29.8 ± 4.2	0.548
	3 d	28.2 ± 3.5	28.3 ± 3.4	0.865

*Hb* hemoglobin, *HCT* hematocrit

**Table 5:** Pain and hip function in two groups (Mean ± SD)

Parameters	Timings	SuperPath group (n = 57)	Conventional group (n = 56)	*p* Value
VAS (score)	Pre	7.19 ± 0.72	7.16 ± 0.66	0.803
	1 d	5.89 ± 0.72	6.46 ± 0.95	0.001*
	3 d	5.12 ± 0.71	5.89 ± 0.73	0.000*
	7 d	4.47 ± 0.68	4.84 ± 0.73	0.007*
	1 m	1.93 ± 0.53	2.34 ± 0.55	0.000*
	3 m	1.11 ± 0.67	1.20 ± 0.62	0.454
	6 m	0.91 ± 0.71	0.89 ± 0.68	0.883
	12 m	0.72 ± 0.65	0.69 ± 0.63	0.850
HHS (score)	7 d	74.41 ± 3.10	69.41 ± 3.38	0.000*
	1 m	84.19 ± 3.08	79.48 ± 3.10	0.000*
	3 m	89.90 ± 2.33	86.25 ± 3.04	0.000*
	6 m	90.68 ± 1.59	90.75 ± 2.41	0.868
	12 m	91.72 ± 1.20	91.67 ± 0.94	0.800

*p < 0.05 indicate significant differences from the conventional. *VAS* visual analog scale, *HHS* Harris hip score

**Table 6:** Radiologic evaluation of the acetabular cup positioning in two groups (Mean ± SD)

Parameters	SuperPath group (n = 57)	Conventional group (n = 56)	*p* Value
Abduction angle (degrees)	43.81 ± 3.08	42.80 ± 3.62	0.131
Anteversion angle (degrees)	14.76 ± 3.26	14.10 ± 2.97	0.262

## Discussion

This study found that, compared with the modified Hardinge approach, patients treated with the SuperPath approach had a shorter operation time, shorter surgical incisions, less muscle damage, less postoperative pain, earlier discharge from the hospital and better postoperative function. However, there was no significant difference between the two surgical approaches in terms of the blood transfusion rate, postoperative complications, abduction angles, anteversion angles, or long-term function. Some femoral neck fracture patients have been selected for THA using the SuperPath approach in our hospital since August 2016. In reviewing our first 45 consecutive SuperPath cases, we discovered that the operation time was longer and fluctuated significantly in the first 25 cases, while the operation time showed a clear downward trend in approximately 25–45 cases. This result is similar to the learning curve of the SuperPath approach previously reported in a retrospective clinical study by Rasuli and Gofton [34]. The results showed that operative time in the SuperPath approach was still decreasing and proficiency continued to improve in the first 50 cases.

Our research results show that the operation time of the SuperPath group was shorter than that of the conventional group. The reason may be that the surgical incision exposure of the modified Hardinge approach was relatively troublesome and the suture time was long. Some previous studies have compared the operation time of the SuperPath approach with the conventional approach, and the results were not consistent (Table 7) [20–29]. The mean operation time of SuperPath approach in these studies was approximately 100 min, which was considerably longer than the average operation time in our study. We think that the reason for this difference may be due to the fact that we had already completed 50 cases of SuperPath learning during the comparative study. We believe that the operation time of the SuperPath approach is no longer than that of the conventional approach when the learning curve of the SuperPath approach reaches a steady plateau. Several randomized controlled studies of SuperPath approach compare with modified Hardinge approach were published in Chinese [35–38]. Huang et al. [36] reported that the operation time was shorter in the SuperPath group (67.4 ± 9.5 min) than the Hardinge group (71.9 ± 5.1 min), which was in agreement with our findings. Another study by Yan et al. [35] reported the longer operation time in the SuperPath group (52 ± 5 vs. 36 ± 15 min). Of course, we cannot ignore the influence of surgical surgeons and clinicians. Notably, the average incision length in the SuperPath group in our study was 5.8 ± 0.4 cm, which was not within the range of the 6–8 cm incision lengths previously reported [16]. The reason may be that the BMIs of Chinese individuals are generally lower than that of foreigners. When there is more fat tissue or more muscle in the hip, a longer surgical incision is needed to expose the surgical field. Except from the benefits to the patient, surgery should also facilitate the surgeon’s operation.

**Table 7:** Comparison of the operation time with additional studies

Study	Diagnosis	Mean operation time (min)	
		SuperPath	Conventional approach
Xie 2017 [20]	OA	104	107
Xu 2019 [21]	FNF	77	81
Meng 2019 [22]	ONFH	103	67
Wang 2020 [23]	FNF	108	102
Jiang 2020 [24]	FNF, OA and ONFH	114	87
Tottas 2020 [25]	OA and ONFH	108	80
Meng 2021 [26]	OA	103	66
Cecere 2021 [27]	FNF	82	81
Hu 2021 [28]	FNF, OA and ONFH	101	128
Li 2021 [29]	FNF and OA	83	64

*FNF* femoral neck fracture, *OA* hip osteoarthritis, *ONFH* necrosis of femoral head

Serum markers, mainly CRP, ESR and CK levels, are widely used to evaluate soft tissue damage in THA [39–41]. The results showed that the levels of CK at different time points in the SuperPath group were obviously lower than those in the conventional group, which confirmed that the SuperPath approach for THA caused less muscle damage and less traumatic inflammation. The SuperPath approach retains the muscles around the hip joint such as the external rotation muscle group, piriformis muscle, gluteus minor muscle, and gluteus medius muscle, ensuring the integrity of the front and rear of the hip capsule [42]. The surgery uses in situ resection of the femoral neck and femoral head instead of the dislocation of the hip joint, so it has the characteristics of reduced trauma and high safety [17].

The total blood loss (TBL) of THA consists of the visible blood loss (VBL) of the intraoperative blood loss, postoperative drainage, and hidden blood loss (HBL) in the tissue [43–45]. In our experiment, the intraoperative blood loss, postoperative drainage volume, HB level and HCT level were selected for comparison between two groups. Our results showed that there was no significant difference in HB and HCT levels between the two groups at any time, while the intraoperative blood loss and postoperative drainage differed between the two groups. We believe the reason for the lower VBL in the SuperPath group was less muscle damage. Although HBL was not specifically calculated, we collected the changes in the HB and HCT levels and inferred that the HBL of the two groups was similar. In a recent retrospective study, the HBL of the SuperPath group was significantly higher than that of the Moore group, but there was no significant difference in TBL between the two groups [21]. The author believes that the reason for the higher HBL in the SuperPath group is because the trabecular expansion procedure destroys the trabecular structure to increase the intramedullary hemorrhage of the femur and the inadequate hemostasis caused by the lower visualization of the surgical field. We believe that after mastering the SuperPath approach, the above two reasons for increased HBL can be properly avoided. Therefore, more prospective RCTs are needed to more appropriately evaluate HBL of SuperPath approach.

In the context of accelerated rehabilitation, minimally invasive THA, including the SuperPath approach in THA, has received increasing attention from joint surgeons [46]. The great advantages of the SuperPath approach are the early recovery of joint function, the shortening of the postoperative hospital stay and promoting rehabilitation [47]. Our results showed that compared with the modified Hardinge approach, the SuperPath approach had a lower VAS score for early pain after THA, shorter postoperative hospital stay, and faster early functional recovery, in agreement with Shen et al. [37] and Yin et al. [38]. One reason that explains the mild postoperative pain symptoms in the SuperPath approach might be that the SuperPath approach is performed through the muscle gap and that the lower limb stretch and twist operations are gentler, which creates conditions for early rapid recovery of patients. A study by Jianbo et al. [48]. also showed that the VAS score, HHS and Barthel Index in the SuperPath approach group at 1-week follow-up intervals were significantly lower than those in the posterolateral approach group but were not significantly different at the 3-month and 2-year follow-up intervals. A recently published 2022 meta-analysis by Ramadanov et al. with 14 studies and 1021 patients showed that The HHS values in the SuperPath were significantly higher than those in the conventional at the 3-, 6- and 12-month (Mean differences (MDs) = 2.4, 95% CI 0.6–4.2; MD = 2.1, 95% CI 0.6–3.6; MD = 0.7, 95% CI 0.1–1.3; respectively). With a few exceptions, all RCTs showed better results for SuperPath in HHS than for conventional. The above research results suggest that the SuperPath approach for THA is conducive to the early functional rehabilitation of patients, but the long-term results need to be confirmed by further studies.

A long-standing principle regarding the position of the components is that the cup abduction angle and anteversion angle are 30°–50° and 5°–25°, respectively [49]. Thus, the aim is to achieve a combined anteversion of 25°–45°, with a range between 25°–35° for men and 30°–45° for women, which represents a ‘‘safe zone’’ defined by Lewinnek to minimize dislocation after primary THA [33]. In the SuperPath group, the abduction and anteversion angles were 43.85° ± 3.01° and 14.76° ± 3.26°, respectively, which were not significantly different from those of the traditional group in our study. One reason that explains this difference is that the SuperPath approach involves reaming and using in situ resection of the femoral neck and femoral head instead of the dislocation of the hip joint. This allows precise measurement of the patients' anteversion and abduction. Recently, Della et al. [46] demonstrated that the cup abduction angle and anteversion angle were safe in the SuperPath approach for THA, and this was confirmed by the study of Xie et al. [20] in an issue of the Journal of Orthopaedic Surgery and Research. These authors believe that such consistency in implant positioning may be the result of a lack of external soft-tissue forces during preparation and implantation and the SuperPath approach using a lateral position. Recent studies have pointed out that the Lewinnek “safe zone” does not truly represent a safe area, and there is still a risk of dislocation in this area [49–52]. They noted that the ideal cup location for some patients may be outside the Lewinnek safe zone, and more advanced analysis is needed to determine the correct target in this subgroup [49, 53]. Considering the functional safe zone defined by Tezuka et al. [52], we also routinely performed flexion (100°–120°), extension (5°–10°), internal rotation (30°–40°) and external rotation (30°–40°) of the patient's limbs after inserting the prosthesis to better evaluate the safety of the prosthesis.

A previous study describing the outcome characteristics for the SuperPath approach showed that the rate of prosthesis dislocation was 0.8%, the rate of deep vein thrombosis was 0.2% and the rate of periprosthetic fracture was 0.8% [19]. Therefore, we believe that SuperPath surgery is safe and useful. As the number of operations gradually increases, a surgeon’s experience is constantly enriched and the incidence of complications is also reduced.

There are several limitations of this study, including the short follow-up time and insufficient number of cases, and the long-term clinical efficacy of the SuperPath approach for THA needs further observation. Meanwhile, the psychological suggestion of patients in the SuperPath group may had an impact on the postoperative pain of VAS score and postoperative rehabilitation exercise. Furthermore, we did not perform gait analysis, which can well reflect the recovery of hip power. In addition, like other minimally invasive approach, the SuperPath approach also requires a relatively long learning curve. During this period, this approach may not be the first choice for elderly patients with femoral neck fractures for young surgeons. A previous study demonstrated that BMI may affect the exposure of surgical incisions and postoperative incision infections [40]. In this study, we included only patients with BMIs less than 30, so our results may be relatively limited. Since the control group did not receive other minimally invasive surgical methods, it may be easy to exaggerate the characteristics of the SuperPath approach for THA. Considering this problem and combining our research results, we will compare the SuperPath approach with the direct anterior approach in follow-up work, which may better reflect the SuperPath approach and provide further value for the clinical setting.

We believe that the SuperPath approach for THA attracts surgeons to choose this approach has these aspects: First, The SuperPath approach reaches the surgical site completely through muscles gap, which has the advantages of less damage, less bleeding, less postoperative pain symptoms, and faster recovery. These points have been confirmed in recent articles and our research. Second, the SuperPath surgery requires many special instruments and the incision is not completely exposed which is the restrictive reason for SuperPath approach promotion, while with the accumulation of experience and the improvement of surgical cooperation, the surgical approach will become more mature, accurate and safe. In addition, the SuperPath approach has many similar steps to the traditional posterolateral approach, so the learning process for orthopedics may be shorter than other minimally invasive approaches. Third, compared with direct anterior approach, the SuperPath approach may be less prone to fracture of the proximal femur because there is no leverage in the process of in-situ femoral bone marrow cavity preparation and femoral neck amputation in elderly patients with severe osteoporosis. This point needs to be proven by subsequent clinical studies.

## Conclusions

In summary, the data from this study illustrate that the SuperPath approach is an effective surgical approach for the treatment of femoral neck fractures in the elderly. Compared with the conventional group, the SuperPath group had a shorter operation time, shorter surgical incisions, less muscle damage, less postoperative pain, an earlier discharge from the hospital and better postoperative function.

## Data Availability

All data generated or analyzed during this study are included in this published article.
